# Metabolomics Signature and Potential Application of Serum Polyunsaturated Fatty Acids Metabolism in Patients With Vitiligo

**DOI:** 10.3389/fimmu.2022.839167

**Published:** 2022-02-10

**Authors:** Zhubiao Ye, Jianru Chen, Pengran Du, Qingrong Ni, Baizhang Li, Zhe Zhang, Qi Wang, Tingting Cui, Xiuli Yi, Chunying Li, Shuli Li

**Affiliations:** ^1^ Department of Dermatology, Xijing Hospital, Fourth Military Medical University, Xi’an, China; ^2^ Department of Dermatology, The Medical Center of Air Force of People’s Liberation Army, Forth Military Medical University, Beijing, China

**Keywords:** metabolomics, arachidonic acid, alpha-linolenic acid, arachidonic acid metabolic pathway, serum, vitiligo

## Abstract

Vitiligo is a depigmented skin disorder caused by a variety of factors, including autoimmune, metabolic disturbance or their combined effect, etc. Non-targeted metabolomic analyses have denoted that dysregulated fatty acids metabolic pathways are involved in the pathogenesis of vitiligo. However, the exact category of fatty acids that participate in vitiligo development and how they functionally affect CD8^+^ T cells remain undefined. We aimed to determine the difference in specific fatty acids among vitiligo patients and healthy individuals and to investigate their association with clinical features in patients with vitiligo. Serum levels of fatty acids in 48 vitiligo patients and 28 healthy individuals were quantified by performing ultra-performance liquid chromatography-tandem mass spectrometry. Univariate and multivariate analyses were carried out to evaluate the significance of differences. Moreover, flow cytometry was used to explore the effect of indicated fatty acids on the function of CD8^+^ T cells derived from patients with vitiligo. We demonstrated that serological level of alpha-linolenic acid (ALA) was markedly upregulated, while that of arachidonic acid (ARA), arachidic acid (AA) and behenic acid were significantly downregulated in patients with vitiligo. Moreover, ALA levels were positively associated with vitiligo area scoring index (VASI) and ARA was a probable biomarker for vitiligo. We also revealed that supplementation with ARA or nordihydroguaiaretic acid (NDGA) could suppress the function of CD8^+^ T cells. Our results showed that vitiligo serum has disorder-specific phenotype profiles of fatty acids described by dysregulated metabolism of polyunsaturated fatty acids. Supplementation with ARA or NDGA might promote vitiligo treatment. These findings provide novel insights into vitiligo pathogenesis that might add to therapeutic options.

## Introduction

Vitiligo is an autoimmune skin disorder that presents as progressive depigmentation of skin due to the destruction of epidermal melanocytes caused by abnormal activation of CD8^+^ T cells (1). Multiple factors like metabolic abnormality, oxidative stress and phenolic compounds exposure were related to the pathogenesis of vitiligo (2–5). Growing evidence suggests that fatty acid metabolism is closely associated with autoimmune diseases, such as psoriasis (6), systemic lupus erythematosus (SLE) (7) and rheumatoid arthritis (RA) (8). Besides, it is reported that the incidence of metabolic syndromes in patients with vitiligo is significantly higher than that in healthy controls (9). However, the in-depth knowledge of vitiligo, in particular on the basis of metabolic dysregulation, remains unclear and need further exploration.

Metabolomics is an emerging field that be used to monitor the alternations in all low molecular metabolites produced by cellular processes, which delineates an overall metabolic profiles have widely applicable to investigate clinical features of various diseases. Recently, the metabolome based on miscellaneous samples, including serum, plasma, blister liquid and urine so on, have revealed that metabolic disturbance of amino acids and lipid mediators are involved in physiological and pathological changes in vitiligo patients (10–13). However, almost all previous studies on the basis of non-targeted metabolomics techniques, which the accuracy of quantitative methods are necessary to promote further. What’s more, most of the research only focuses on the alternation of metabolites and dysregulated metabolic pathway, their specific role in the development and treatment various disorders still needed to further investigation. Therefore, targeted metabolomics method and confirmation experiments are of great significance to improve the accuracy of altered metabolites and to promote the management of vitiligo patients.

Several previous studies reported that supplementation with EPA and DHA contributes to the recovery of autoimmune diseases like RA and SLE (14–16). Another research disclosed that supplementation with ARA or aspirin, COX-2 inhibitor, also prevents healthy individuals from diabetes mellitus (17). What’s more, growing evidence supports that omega-3 polyunsaturated fatty acids are conducive to alleviate CD8^+^ T cell-mediated inflammatory response in various disorders (18, 19). Our previous non-targeted metabolomics study also found that levels of fatty acids manifested the most alterations among that of various metabolites. More importantly, the enriched Kyoto encyclopedia of gene and genomes (KEGG) pathway analysis revealed that several fatty acid metabolic pathways were significantly associated with vitiligo development (20). However, which specific fatty acid plays a key role and how it works in vitiligo remains ill-defined.

In our study, we conducted a targeted metabolomics assay to assess serum FAs concentration and demonstrated the major differentially expressed fatty acids in vitiligo, and further evaluated their correlation with clinical features. Additionally, we investigated the effect of ARA or its metabolic pathway on the activation and effect function of CD8^+^ T cells.

## Materials and Methods

### Study Subjects

Serum for fatty acids profiles was derived from a continuous of sample of 76 participants: 48 vitiligo patients and 28 age- and gender-matched healthy controls. The diagnosis of vitiligo was ascertained and VASI scores assessed by dermatologists. Corresponding healthy volunteers were recruited from physical examination center of Xi Jing hospital to undertake the same testing as vitiligo patients. All study participants with active autoimmune diseases, malignancies, diabetes mellitus, pregnant and breastfeeding women were excluded from this study. Besides, study subjects who took glucocorticoids, antibiotics and other drugs or diet habit that might affected fatty acid metabolism in the past three months were ruled out as well. Epidemiological data including gender, age, disease duration, BMI, disease activity, clinical presentations, treatments, was face-to-face assessed and recorded by dermatologists. The VASI scores were evaluated by two senior dermatologists and calculated by average. Laboratory measurements including TC, TG, HDL-C and LDL-C were measured or collected from electronic patient record in hospital.

### Chemicals and Reagents Used for Metabolomics Analysis

Detailed information of chemicals and reagents was described in the **Supplementary Data**.

### Sample Preparation and Fatty Acid Extraction

Serum samples were obtained by centrifugation of fresh blood samples for 5 min at 2500 rpm at 4°C and immediately quenched by liquid nitrogen and stored at -80°C until further analyses. Prior to the assay, serum samples were left at -20°C for 30 minutes and then thawed on ice-bath to diminish sample degradation. All fatty acid standards were prepared to storage solution at 5.0 mg/mL. An appropriate amount of individual stock solution was mixed to obtain working standard solutions, which were made to produce the calibration curves. The fatty acid extraction method followed by a previously described report (21, 22). Briefly, 20 μl of serum or each standard solution were mixed with 120 μl cold methanol with internal standard solution were added to a 96-well plate. The mixed samples were centrifuged at 4000 × g at 4°C for 30 min. An aliquot of 30 μl the supernatant was transferred to a new 96-well plate. Subsequently, 20 μl of derivative reagent, 3-nitrophenylhydrazine (3-NPH) was added to each well, the plate was locked and the derivatization was performed at 30°C for 60 min. After derivatization, 400 μl of ice-cold 50% methanol solution was added and stored at -20°C for 20 minutes and followed by 4000 × g centrifugation at 4°C for 30 minutes. Finally, the supernatant was used for LC-MS analysis.

### Analysis of Metabolites by UPLC-MS/MS

Metabolomics profiling was performed using a UPLC-MS/MS platform (Acquity UPLC-Xevo TQ-S; Waters). The samples were randomized and analyzed using an Acquity UPLC BEH C18 1.7 μM VanGuard pre-column (2.1 × 5 mm) and analytical column (2.1 × 100 mm). The column temperature and sample manager temperature were maintained at 40°C and 10°C, respectively. Water with 0.1% formic acid (solvent A) and acetonitrile/IPA (70:30) (solvent B) were used as mobile phases. The flow rate and injection volume were 400 μl/min and 5 μl, respectively. The gradient condition was scheduled as follows: 0-1 min (50% B), 1-3.5 min (50-78% B), 3.5-12.2 min (78-100% B), 12.2-14.2 min (100% B), 14.2-14.5 min (100-50% B), 14.5-16 min (50% B). The mass spectrometer was operated in the negative mode with a 2.0 kV capillary voltage. The source and desolvation temperatures were 150°C and 550°C, respectively. And the desolvation gas flow was 1200 L/hr.

All samples were analyzed at beginning and end of each batch run and data for each ionization technique were gained in negative ion mode. The quality control (QC) samples were prepared by mixing equal volume of each serum sample (48 vitiligo samples, 28 healthy control samples) and then injected at regular intervals (after every 14 test samples for LC-MS) throughout the analytical run. Reagent blank samples consist of high purity solvents were randomly inserted among the real sample queue to serve as a useful alert to systematic contamination, as well as wash the column and remove cumulative matrix effects throughout the study.

### Analytical Validation

The method validation was performed by the following ways: linearity and quantification limits, reproducibility and recovery of result. The detailed information are presented in **Supplementary Data** and **Supplementary Table S1**.

### Cell Separation and Culture

Peripheral blood samples were collected by EDTA vacutainer tubes. Peripheral Blood Mononuclear Cells (PBMCs) were separated from the fresh blood samples of vitiligo patients by density-gradient centrifugation with lymphocyte separation medium. For CCK-8 assay, the CD8^+^ T cells were positively isolated from PBMCs with a magnetic-activated cell sorting CD8^+^ T Cell Isolation Kit (Miltenyi Biotec) according to the manufacturer’s instructions. Cells were cultured in RPMI 1640 (Gibco) supplemented with 10% fetal bovine serum (Gibco), and 1% penicillin-streptomycin solution.

### CCK-8 Assay

The effect of ARA on the survival of human naïve CD8^+^ T cells were measured using the CCK-8 assay based on the manufacturer’s instruction. Briefly, the isolated CD8^+^ T cells were planked with (10^6^ cells mL^-1^) at 96-well U-plate and incubated in the presence of different concentrations (0, 5, 10, 20, 50, 100 μM) of ARA for 48 h. The viability of CD8^+^ T cells was detected by the CCK-8 assay and the Optical Density was measured at 450 nm with a microplate reader.

### Flow Cytometry Analysis

The detailed procedures of flow cytometry analysis are provided in **Supplementary Data**.

### Data Processing and Statistical Analysis

In LC-MS/MS-based metabolomics analysis, the targeted raw data files were processed through using the MassLynx software (v4.1, Waters, Milford, MA, USA) to perform peak integration, calibration, and quantitation for each metabolite. The powerful package R studio and MetaboAnalyst 5.0 (a web-based software tool for metabolomic data analysis, https://www.metaboanalyst.ca/) were used for statistical analyses. To reduce the differences on the concentration of fatty acid between samples, auto-scaling was performed to make sure each variable comparable to each other. For multivariate statistical analysis, PLS-DA was used to visualized the difference between global metabolic profiles among the given groups that provides more valuable information, and RF algorithm was applied to enhance the quality of multivariate analyses and to avoid the risk of overfitting. For univariate analysis, discrete variables were summarized in percentage and compared between different groups using Chi-Square test or Fisher’s exact test. Shapiro-Wilk test was applied to evaluate the distributions of continuous variables. Student’s *t*-test or Mann-Whitney *U* test were implemented to determine the statistical significance of each fatty acid, according to the distribution type of data. Pearson correlation analysis was carried out for clarifying the association between fatty acid and clinical characteristics.

ROC curves were performed to assess the diagnostic performance of fatty acids by MetaboAnalyst 5.0 and Graph Prism 8.0. Supporting vector machine and 10-fold cross-validation was established to avoid the problem of potential over-fitting in producing combined predicted ROC curves. Binary logistic regression was used to evaluate the risk factor of vitiligo.

For determining the potentially altered metabolic pathways in vitiligo, pathway analysis was carried out by MetaboAnalyst 5.0. We evaluated altered fatty acids within a pathway and its function of the pathway through variations in pivotal junction points of the pathway.

## Results

The anthropometric and clinical features of study subjects were presented in **Supplementary Table S2**. Samples for targeted metabolomics analysis were donated by a total of 48 vitiligo patients (24 males and 24 females, median and IQR age (34.5, 25.75-43.75) years) and 28 healthy controls (11 males and 17 females, median and IQR age (35, 26.5-40.75) years). The average VASI of patients with vitiligo was 1.76. There were no significant differences in gender, age, body mass index (BMI), total cholesterol (TC), triglyceride (TG), high density lipoprotein cholesterol (HDL-C), or low density lipoprotein cholesterol (LDL-C), between the vitiligo group and healthy control group.

### Fatty Acid Metabolic Profiles of Vitiligo Patients Differed From That of Healthy Controls

**Figure 1:**
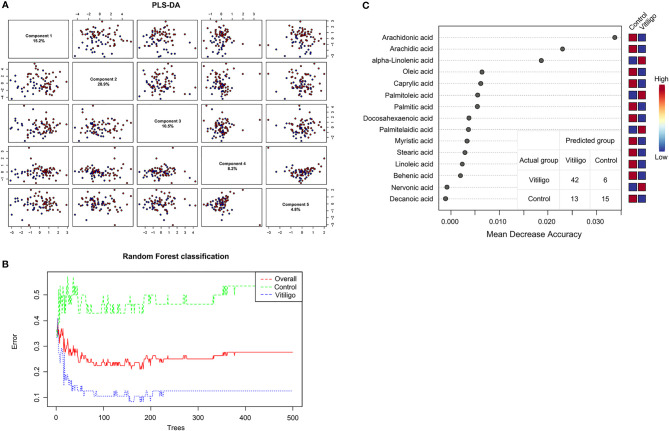
Utilization of fatty acid profiles for classification of vitiligo patients and healthy controls by PLS-DA and RF analysis. **(A)** PLS-DA model shown the five principal components with the relative variances in pairwise scores plots. **(B)** Cumulative error rates by RF Classification. The red line represents the overall error rate; the vitiligo error rate and control error rate is shown as the blue line and green line, respectively. **(C)** Significant features confirmed by RF. The features are ranked by the mean decrease in classification accuracy when they are permuted. PLS-DA, partial least squares discriminant analysis; RF, random forest.

To clarify the connection between fatty acid metabolism and vitiligo, targeted metabolomics based on ultra-performance liquid chromatography-tandem mass spectrometry (UPLC-MS/MS) was applied on serum samples from 24 active vitiligo patients, 24 stable vitiligo patients and 28 healthy controls. Serological concentrations of 19 fatty acids of the cohort were shown in **Supplementary Figure S1**. Multivariate statistical analysis was carried out on the fatty acid metabolic profiles to demonstrate whether there were characteristic alterations that distinguished vitiligo patients from healthy controls. According to partial least squares discriminant analysis (PLS-DA), although principal components 1-5 elucidated 66.5% of the variance of the LC-MS/MS data, there was no clear clustering that could distinguish vitiligo patients from healthy controls (**Figure 1A**). Moreover, the low R2 and Q2 values (R2 = 0.36, Q2 = 0.06), which both indicated the quality of the PLS-DA model, suggested that the model was overfitted. Sequentially, to improve the diagnostic accuracy based on the fatty acid metabolic profiles, random forest (RF) algorithm was performed to identify similar regions that distinguished vitiligo patients and healthy individuals with an out-of-bag error of 0.276 for categorization (**Figure 1B**). RF analysis generated a series of fatty acids ranged by their importance to the classification program, and the top 15 fatty acids ascertained were shown in **Figure 1C**. Metabolite, especially ARA, was recognized as contributing to the division of groups. To summarize, the fatty acids that explain the difference of metabolic profiles between vitiligo patients and their healthy counterparts were determined.

**Figure 2:**
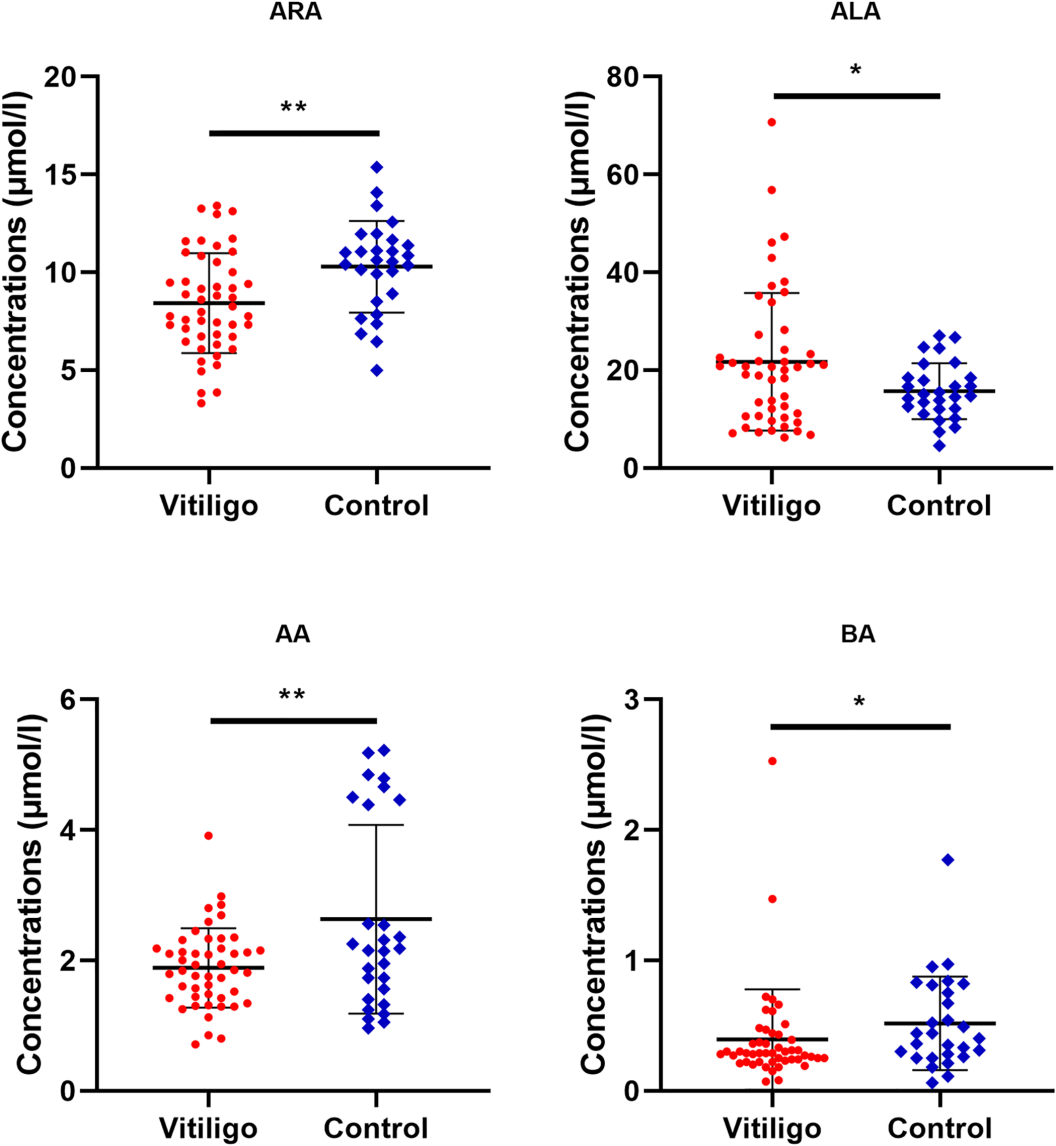
Differential fatty acid levels in vitiligo patients as compared to healthy controls. The levels of ARA, ALA, AA and BA in vitiligo patients or healthy individuals were compared. The mean and standard deviation were represented by middle black line, the lower and upper black lines, respectively. ARA, arachidonic acid; ALA, alpha-linolenic acid; AA, arachidic acid; BA, behenic acid. *P < 0.05, **P < 0.01.

To further clarify which specific fatty acid altered the metabolic profile of vitiligo patients, univariate statistical analysis was performed to hunt for differentially expressed fatty acids. As a result, serological levels of ALA were found to be the most significantly elevated in vitiligo patients compared with healthy controls, and levels of ARA, AA and behenic acid (BA) tended to diminish in the vitiligo group (**Figure 2** and **Supplementary Table S3**). Additionally, binary logistic regression presented in **Supplementary Table S4** revealed that ARA and AA were appreciated as a protective factor, while ALA was evaluated as a risk factor. These findings further specified the exact fatty acids that might be closely associated with vitiligo.

Furthermore, we asked whether some fatty acids could distinguish active vitiligo from stable vitiligo. Both the PLS-DA model and RF algorithm showed no clear differences among all fatty acids measured between progressive vitiligo and stable vitiligo (**Supplementary Figures S2A, B**). This disclosed that there were no marked differences in fatty acids among subgroups.

### ALA Is Positively Correlated With VASI

**Figure 3:**
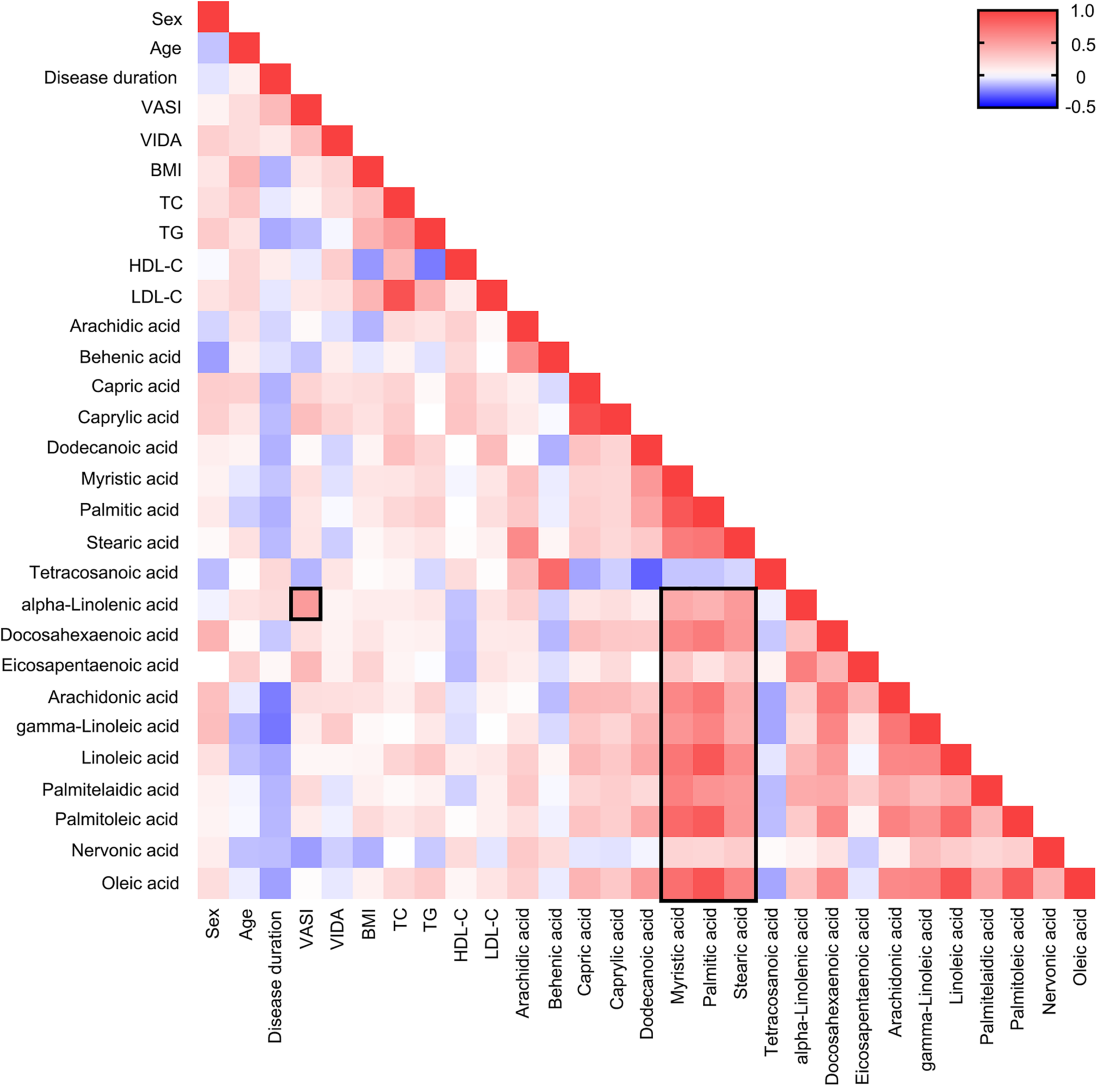
Heat map of Pearson correlation analysis among fatty acids and clinical characteristics. Positive correlations are shown in red and negative correlations are shown in blue. The darker the color, the more relevant.

Factors like sex, age, BMI, TG could influence fatty acids concentration in body fluids, thus the association between fatty acids and clinical characteristics should be considered. Even though strong correlation coefficients were found between long-chain saturated fatty acid (LC-SFA) and other fatty acids, notably, the strong correlation coefficient (Pearson correlation analysis, r = 0.523, *P*< 0.001) was discovered between VASI and ALA presented in **Figure 3**. What’s more, a stronger correlation coefficient was uncovered between VASI and ALA (r = 0.692, *P*< 0.001). These results indicated that the concentrations of some fatty acids, such as ALA, might partly reflect the severity of vitiligo.

### ARA Is the Most Characteristic Biomarker and Arachidonic Acid Metabolism Is the Most Enriched Metabolic Pathway in Vitiligo

**Figure 4:**
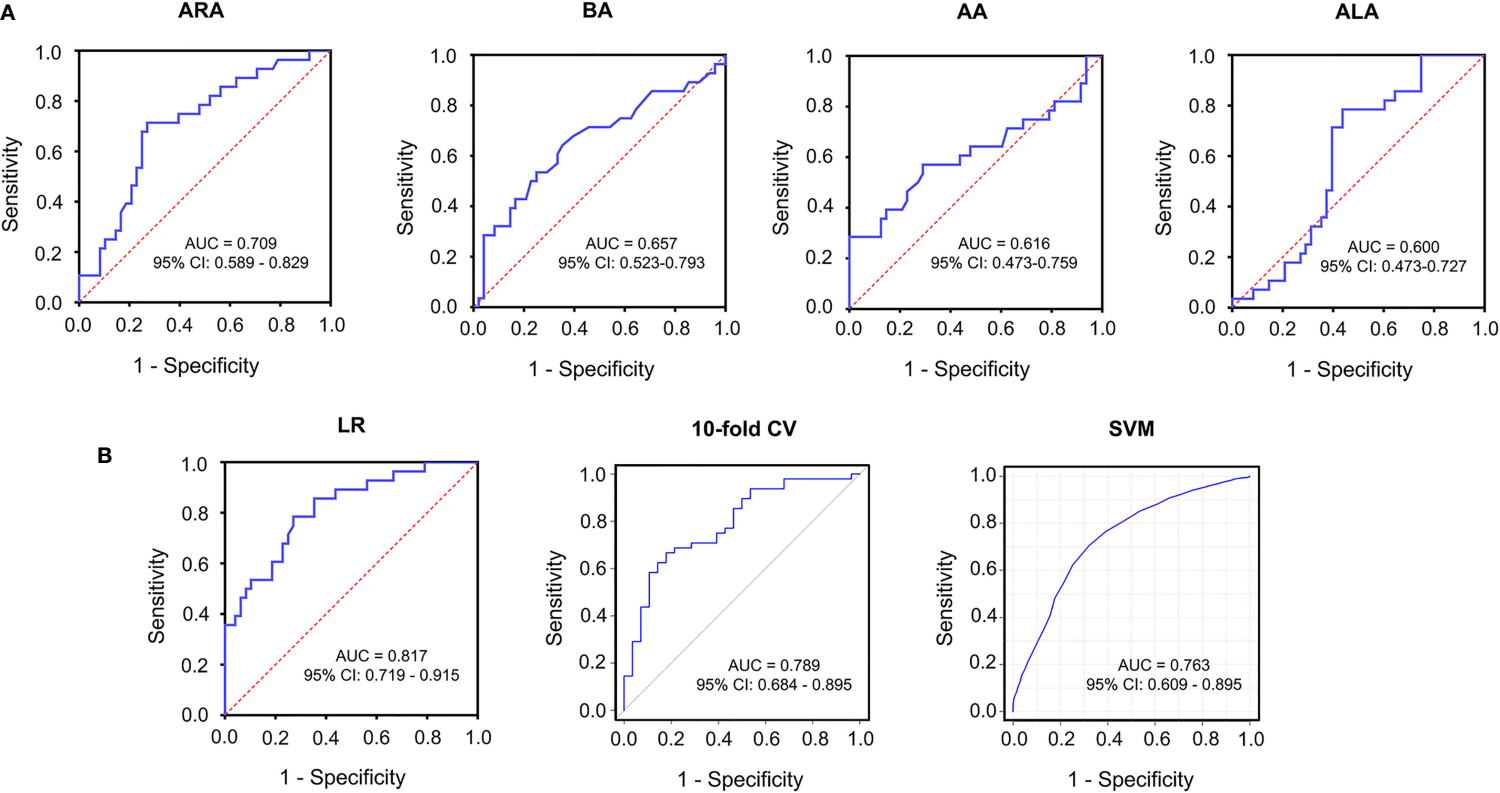
Receiver operating characteristics curve for significantly altered serum metabolites in the comparison between vitiligo patients and healthy individuals. **(A)** ROC curves of ARA with AUC = 0.709, BA with AUC = 0.657, AA with AUC =0.616, ALA with AUC = 0.600. **(B)** ROC curves of combined models selected from four differential fatty acids using LR (AUC = 0.817), 10-fold CV (AUC = 0.789), SVM (AUC = 0.763), respectively. ROC, receiver operating characteristics; AUC, area under the curve; LR, logistic regression; 10-fold CV, 10-fold cross validation; SVM, supporting vector machine; CI, confidence interval.

The diagnostic efficacy of significantly altered fatty acids was assessed by MetaboAnalyst 5.0. As a result, the area under the curve (AUC) of ARA was 0.709 as shown by the receiver operating characteristic curve (ROC), the AUC of other differentially expressed fatty acids, such as BA, AA and ALA were 0.657, 0.616, and 0.600, respectively (**Figure 4A**). To improve the diagnostic accuracy of vitiligo, a combined predictive model was set up using four differential fatty acids, in which the AUCs were 0.817 (95% confidence interval (CI): 0.719-0.915) with a sensitivity of 78.6% and specificity of 72.9% (**Figure 4B**). In order to make the prediction model more reliable and avoid over-fitting issues, we adopted a machine learning model of a 10-fold cross-validation and supporting vector machine (SVM). The AUC value of the model remained 0.789 and 0.763, respectively (**Figure 4B**). These data indicated that fatty acids metabolic profiles, especially ARA, could be utilized to differentiate vitiligo patients from healthy individuals. Metabolic pathways are also critical in characterizing the metabolic profile of a disease in addition to metabolites. According to KEGG metabolic database disturbed arachidonic acid metabolism, alpha-linolenic acid metabolism and linoleic acid metabolism were markedly enriched (**Supplementary Figure S3**). Moreover, the FDR < 0.05 of arachidonic acid metabolism denoted that it plays a critical role in patients with vitiligo.

### ARA Inhibits the Proliferation and Activation of CD8^+^ T Cells *In Vitro*

**Figure 5:**
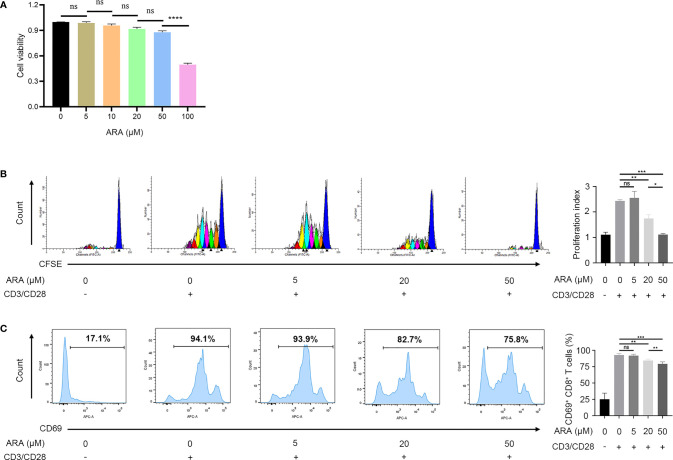
ARA inhibited the proliferation and activation of CD8^+^ T cells in a concentration-dependent manner. **(A)** The cell viability of CD8^+^ T cells on ARA by CCK-8 assay (n = 3). **(B)** Flow cytometric analysis of CD8^+^ CFSE^+^ labeled T cells in vitiligo PBMCs after stimulation with anti CD3/CD28 for 5 days with ARA 0 to 50 μM (n = 3). **(C)** Flow cytometric analysis of CD8^+^ CD69^+^ labeled T cells in vitiligo PBMCs after stimulation with anti CD3/CD28 for 1 days with ARA 0 to 50 μM (n = 6). ARA, arachidonic acid; μM, μmol/l; ns, no significance, *P < 0.05, **P < 0.01, ***P < 0.001, ****P < 0.0001.

Since ARA was significantly altered in vitiligo and served as a protective agent, we speculated that ARA supplementations might ameliorate the autoimmunity in vitiligo. Therefore, we focused on the influence of ARA on CD8^+^ T cells in patients with vitiligo *in vitro*. Firstly, the cell cytotoxicity assay indicated that there were no significant alterations in cell viability when the concentrations of ARA were under 50 μM (**Figure 5A**). Thus, we chose 50 μM as a limit concentration of ARA in our experiments *in vitro*.

Our result showed that ARA inhibited human CD8^+^ T cells proliferation stimulated by CD3/CD28 monoclonal magnetic beads in a concentration-dependent manner, and the proliferation of CD8^+^ T cells was almost completely suppressed at 50 μM (**Figure 5B**). Further, the expression of CD69 on CD8^+^ T cells was concentration-dependently inhibited at concentrations higher than 20 μM (**Figure 5C**). These results indicated that ARA could inhibit the activation and proliferation of CD8^+^ T in a concentration-dependent manner.

### ARA and 5-LOX Inhibitor Suppress the Expression of Effector Molecules of CTLs

**Figure 6:**
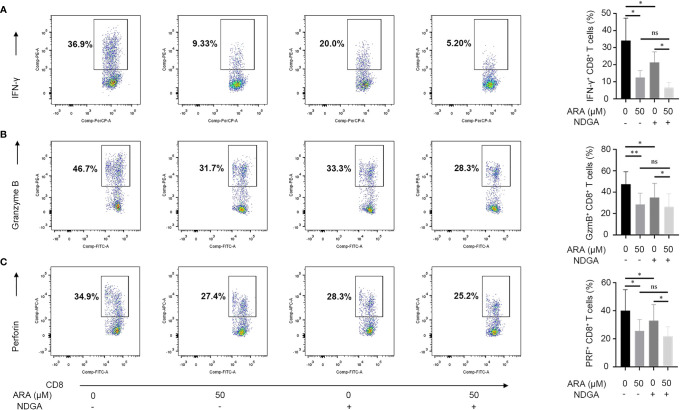
ARA and NDGA inhibit effector functions of CD8^+^ T cells. Vitiligo PBMCs were stimulated with anti-CD3/CD28 for 48 hours in different conditions (with or without NDGA (10μM) in the presence or absence of ARA (50μM)) for 48 h. Expression of IFN-γ **(A)**, Granzyme B **(B)**, and Perforin **(C)** in CD8^+^ T cells was determined by flow cytometry (n = 6). ARA, arachidonic acid; NDGA, nordihydroguaiaretic acid; μM, μmol/l; ns, no significance, *P < 0.05, **P < 0.01.

We continued to clarify the effect of ARA on the expression of CD8^+^ T effector molecules. Results disclosed that ARA significantly suppressed the expression of IFN-γ, granzyme B and perforin (**Figures 6A–C**). Thus, ARA might inhibit the effector function of CD8^+^ T cells.

Considering that cyclooxygenase (COX) pathway and lipoxygenase (LOX) pathway both participate in the arachidonic acid metabolism, we further employed celecoxib, COX-2 inhibitor, and NDGA, 5-LOX inhibitor, to specify the signaling pathway that contributes to ARA-mediated functional suppress of CD8^+^ T cells. Our results showed that NDGA, instead of celecoxib, could inhibit the expression of IFN-γ, granzyme B and perforin (**Figures 6A–C** and **Supplementary Figures S4A–C**). Furthermore, we found the synergistic administration of ARA and NDGA performed better than their separate use in inhibiting the expression of IFN-γ, granzyme B and perforin in CD8^+^ T cells activated by CD3/CD28 monoclonal magnetic beads. Collectively, these results indicated that ARA and 5-LOX inhibitor might inhibit the expression of effector molecules in CD8^+^ T cells *in vitro*.

## Discussion

In the present study, we found a significant increase of ALA and decrease of ARA in vitiligo patients, and that supplementation with ARA or NDGA might suppress the self-reactive CD8^+^ T cells, characterized by the inhibition of the proliferation and activation of CD8^+^ T cells and lower expression levels of IFN-γ, granzyme B and perforin.

Circulating free fatty acids like ALA, EPA and ARA, are reportedly related to several autoimmune diseases. We evaluated the concentration of free fatty acids and observed increased levels of ALA, decreased levels of AA and BA in the serum of patients with vitiligo (*P* < 0.05). Unlike our results, Siro et al. discovered that the serum level of ALA was significantly reduced in patients with active vitiligo, while the expression of AA and BA were not markedly changed (23). The contradictory results might be attributed to either limited sample size or distinct measured methods in different studies, and the targeted metabolomics methods used in our study were more accurate than non-targeted data even though correction for signal drift (24).

Besides, the downregulated concentration of serum ARA in vitiligo was observed in our study, which paralleled previous reports (23). The main sources of ARA in the human body are synthesis from linoleic acid by phospholipase (PLA2, PLC and PLD) and supplement by dietary intake, while the disposition was metabolized to ARA-derived lipid mediators, such as prostaglandins (PGs), leukotrienes and epoxyeicosatrienoic acids (EETs). The reason for ARA decrease is still unclear. We surmise that metabolic exhaustion might provide an explanation considering the reportedly elevated levels of PGs (PGE2, PGD2 and PGF2α) in vitiligo lesions and leukotrienes in urine (11, 13), which are both metabolites of ARA. It’s worth noting that abnormality of phospholipase in patients with vitiligo has not been previously reported, which needs further study.

In our present study, we also explored the relationship between the concentration of serum fatty acids and the vitiligo severity and activity. We observed that ALA was positively correlated with the disease severity (VASI scores), suggesting the potential of serum ALA as a new biomarker to assess vitiligo severity. Further pathway analysis disclosed that alpha-linolenic acid metabolism was critical to vitiligo pathogenesis. Previous global metabolomics profiles revealed that alpha-linolenic acid metabolism was significantly up-regulated in RA, and they suggest that long-term inflammation and nonsteroidal anti-inflammatory drugs intake may be responsible for the disturbed alpha-linolenic acid metabolism (25). Our study further validated that alpha-linolenic acid metabolic disturbance might be associated with chronic inflammatory status in vitiligo. Physiologically, alpha-linolenic acid metabolic pathway could be transformed into bioactivate mediators such as EPA and DHA. Thus, the observed positive correlation between ALA and VASI might corroborate with previous reports that EPA was inversely associated with the severity of RA and experimental autoimmune encephalomyelitis on account of the speculated low biotransformation rate of ALA in vitiligo (26, 27). Besides, several clinical experiments have disclosed that supplementation with EPA, DHA and their metabolites, resolvins, could contribute to the recovery of autoimmune diseases, such as diabetes and multiple sclerosis (28–30), which indicated that the role of metabolites of ALA in vitiligo still needs further exploration. Our study also revealed that there were no differences in free fatty acid between active vitiligo and stable vitiligo. The association between fatty acid levels and vitiligo activity still needs further investigation owning to relatively small sample sizes in our research.

Our study also found that ARA was a sensitive biomarker for the predictive model of vitiligo. Although the diagnosis of vitiligo was relatively definitive by clinical observation and Wood’s lamp, ARA, along with other unsaturated fatty acids could help reflect inflammation and autoimmunity status (31, 32). Previous studies confirmed that ARA was strongly associated with the risk of chronic inflammatory diseases and diabetes-associated autoimmunity (33–35). Our study revealed the role of ARA in the pathogenesis and treatment of vitiligo.

Vitiligo is an autoimmune skin disorder mainly mediated by autoreactive CD8^+^ T cells that lead to skin depigmentation. Our results indicated that ARA and its metabolic pathway were closely associated with autoimmune diseases. A preceding study reported that ARA, the terminal metabolite of linoleic acid, could suppress CD8^+^ T cell proliferation and activation in healthy individuals (36), which backed up our findings. Additionally, several pieces of research showed that ARA could inhibit the production of IFN-γ in various diseases, such as metal-induced allergy diseases or neuroinflammatory disorders (37, 38). In our present study, we firstly demonstrated that ARA could inhibit the secretion of granzyme B and perforin.

Cyclooxygenase (COX) and lipoxygenase (LOX) metabolic pathway are regarded as the most important pathways in regulating arachidonic acid metabolism. Besides, metabolites of COX-2 and 5-LOX pathway have definitive immunomodulatory effects. A recent study found that celecoxib was effective in relieving swelling and inflammation in accordance with inhibition of IFN-γ production in metal-induced allergy diseases (39). However, our results showed that celecoxib couldn’t influence the expression of IFN-γ, as well as granzyme B and perforin generated by CD8^+^ T cells in vitiligo patients. It may partly result from PGs, the metabolites of COX-2 pathway, because low concentrations of PGE2 potentiate Th1 cells differentiation, while high concentrations work right reversely (40). Whereas NDGA, as a well-known 5-LOX inhibitor, could significantly inhibited the expression of IFN-γ, granzyme B and perforin of CD8^+^ T cells derived from vitiligo patients. In line with our findings, NDGA was previously reported to inhibit IFN-γ-induced inflammatory response *in vitro* (41). In addition, their results suggest that NDGA regulates IFN-γ-mediated inflammation through mechanisms uncorrelated to the inhibition of 5-LOX metabolites. Inhibition of 5-LOX pathway could increase PGE2 generation through upregulating COX-2 pathway (42). And higher concentrations of PGE2 could inhibit the secretion of IFN-γ to play a protective role in vitiligo. Therefore, inhibition of 5-LOX pathway in vitiligo treatment might be therapeutically exploitable.

Despite these findings, our study has several limitations. Firstly, although metabolomics can provide a comprehensive understanding of the disease, it still has some variability and inconsistencies due to the limited sample size (43). And a large number of population studies should be carried out in the future to avoid the limited statistical power. Secondly, our findings were derived from *in vitro* experiments, hence the effect of ARA and 5-LOX inhibitor on CD8^+^ T cells and their capability in promoting repigmentation in vitiligo treatment still need further validation *in vivo*.

In summary, we performed a targeted metabolomics study and provided evidence that metabolic abnormality of fatty acids, especially ALA and ARA, were tightly associated with vitiligo. Moreover, ALA and ARA might be used for assessing vitiligo severity and predicting disease. Finally, our results found that ARA and NDGA could inhibit the function of CD8^+^ T cells, which might offer a novel strategy for the treatment of vitiligo.

## Data Availability Statement

The original contributions presented in the study are included in the article/**Supplementary Material**. Further inquiries can be directed to the corresponding authors.

## Ethics Statement

The studies involving human participants were reviewed and approved by Ethics Committee of Xijing Hospital. The patients/participants provided their written informed consent to participate in this study.

## Author Contributions

ZY, JC, PD, CL, and SL came up with the design of the experiment. All authors contributed to the performance of the assays and acquisition of the sequencing and experimental data. ZY, JC, PD, and SL analyzed the data. ZY, JC, PD, CL, and SL wrote and revised the manuscript with input from all the authors.

## Funding

This work was supported by the National Natural Science Foundation of China (No. 81930087, No. 12126606).

## Conflict of Interest

The authors declare that the research was conducted in the absence of any commercial or financial relationships that could be construed as a potential conflict of interest.

## Publisher’s Note

All claims expressed in this article are solely those of the authors and do not necessarily represent those of their affiliated organizations, or those of the publisher, the editors and the reviewers. Any product that may be evaluated in this article, or claim that may be made by its manufacturer, is not guaranteed or endorsed by the publisher.
