# Supplementary material for: Metabolomics Signature and Potential Application of Serum Polyunsaturated Fatty Acids Metabolism in Patients With Vitiligo
Source: Front Immunol. 2022 Feb 10;13:839167. doi: 10.3389/fimmu.2022.839167 (PMC8866849; doi:10.3389/fimmu.2022.839167)
Supplement: Supplementary file 1 [file DataSheet_1.docx]

**Supplementary Materials & Methods**

**1.1 Chemicals and reagents used for metabolomics analysis**

All of the standards of fatty acids and stable isotope-labeled internal standards were obtained from Sigma-Aldrich (St. Louis, MO, USA), Steraloids Inc. (Newport, RI, USA) and TRC Chemicals (Toronto, ON, Canada). Formic acid was of (Optima grade and obtained from Sigma-Aldrich (St. Louis, MO, USA). Methanol (Optima LC-MS), acetonitrile (Optima LC-MS), and isopropanol (Optima LC-MS) were purchased from Thermo-Fisher Scientific (FairLawn, NJ, USA). Ultrapure water was produced by a Mill-Q Reference system equipped with a LC-MS Pak filter (Millipore, Billerica, MA, USA).

**1.2 Analytical validation** (1)

**Linearity and Quantification Limits.** In order to obtain the calibration curves, we plotted the peak area ratios of metabolites and internal standards against the concentrations of the metabolite. We further determined the linearity of the response by linear regression modeling according to a series of standards at different concentrations in the solvent (Supplementary Table S1). For all the compounds investigated with the ability to detect a wide concentration range, the correlation coefficient value (R^2^) was greater than 0.9900 (Supplementary Table S1). Additionally, the quantification limit of each compound was determined by analyzing the signal-to- noise ratio (S/N) provided by the MassLynx software.

**Reproducibility of Results.** The reproducibility of the automated derivatization technique and the UPLC-TQMS analysis were investigated by using both the standard mixtures and biological serum samples from vitiligo patients and healthy controls. Using successive replicate measurements, we analyzed the prepared standard mixtures and serum samples, respectively. As showed in Supplementary Table S1, most of the test compounds and metabolites identified in serum demonstrated acceptable reproducibility with relative standard deviations (RSDs) less than 15%, excepting some compounds with concentrations close to the quantification detection limit.

**Recovery.** We employed spiking experiments in serum samples to assess the metabolite recoveries. The results of the recovery experiments were shown in Supplementary Table S1. Most metabolites had good accuracy with the recovery ranging from 80% to 120% and CV less than 15%.

**1.3 Proliferation assay**

The influence of ARA on CD8^+^ T cells proliferation were determined by flow cytometry analysis with CFSE labelling. Concisely, the PBMCs were stained with CFSE (2.5 μM) for 15 min at 37℃, stopped by 10% FBS, washed with PBS and resuspended in complete RBMI 1640 medium. Soon afterwards, CFSE-labelled PBMCs were planked at 96-well U-plate and stimulated by CD3/CD28 monoclonal magnetic beads in the presence of different concentrations (0, 5, 20, 50 μM) of ARA for 5 days. The level of CFSE-labelled CD8^+^ T cells proliferation was investigated by flow cytometry after stained with PERCP anti-human CD8a antibody.

**1.4 Activation assay**

The activation of CD8^+^ T cells were characterized with the expression of CD69 by flow cytometry. Briefly, PBMCs were stimulated with or without CD3/CD28 monoclonal beads in the different concentrations (0, 5, 20, 50 μM) of ARA for 24 h. The cells were collected, washed and stained with anti-human CD8a (FITC, biolegend) and anti-human CD69 (APC, biolegend) for 30 min at 4℃. Subsequently, the PBMCs were washed, fixed and determined by flow cytometry.

**1.5 Detection of cytokines**

The measurement of effector molecules of CTLs were characterized by the expression of IFN-γ, granzyme B and perforin. Briefly, the PBMCs were stimulated with or without CD3/CD28 monoclonal magnetic beads in the presence of ARA (50 μM), celecoxib (1 μM) or nordihydroguaiaretic acid (NDGA) (10 μM) for 48 h. Subsequently, PBMCs were collected, washed, fixed and stained with anti-human CD8a (FITC, biolegend), IFN-γ (BV421, biolegend), granzyme B (PE, biolegend) and perforin (APC, biolegend) for 30 min at 4℃. Then, the cells were washed and assessed by flow cytometry.

**Supplementary Reference:**

1. Li J, Sun B, Guo Y, Wang X, Wang Y, Zhang H, et al. A Metabolite Array Technology for Precision Medicine. Anal Chem (2021) 93(14): 5709-5717. doi: 10.1021/acs.analchem.0c04686.

**Supplementary Tables**

**Supplementary Table S1**. MRM transitions of 3-NPH derivatized metabolites, linearity range, precision and accuracy.

Detailed information is displayed in an excel version.

**Supplementary Table S2**. Epidemiological characteristics and clinical parameters of patients with vitiligo and healthy controls

| Variables | Vitiligo (n=48) | Control (n=28) | *P* value |
| --- | --- | --- | --- |
| Epidemiological characteristics |  |  |  |
| Sex, male n (%) | 24 (50) | 11 (39.29) | 0.491^a^ |
| Age (years), mean (median, IQR) |  |  |  |
| Age at blood sampling | 35.25 (34.5, 25.75-43.75) | 35.36 (35, 26.5-40.75) | 0.966^b^ |
| Age of disease onset | 27.90 (25.5, 18-38.5) |  |  |
| BMI (kg/m2), mean (median, IQR) | 22.93 (22.79, 20.31-25.74) | 22.43 (22.01, 20.58-24.67) | 0.521^b^ |
| Disease Duration (months), mean (median, IQR) | 87.42 (57, 24-112.5) |  |  |
| VASI score, mean (median, IQR) | 1.76 (0.5, 0.19-1.95) |  |  |
| Activity, n (%) |  |  |  |
| Active vitiligo | 24 (50） |  |  |
| Stable vitiligo | 24 (50） |  |  |
| Associated comorbid autoimmune disorder, n (%) |  |  |  |
| Thyroid disease | 3 (6.25) |  |  |
| Diabetes mellitus | 2 (4.17) |  |  |
| ulcerative colitis | 1 (2.08) |  |  |
| Clinical parameters |  |  |  |
| TC (mmol/L), mean (median, IQR) | 4.47 (4.57, 3.85-5.14) | 4.42 (4.31, 4.08-4.93) | 0.782^b^ |
| TG (mmol/L), mean (median, IQR) | 1.43 (1.11, 0.77-1.85) | 1.28 (0.98, 0.77-1.46) | 0.549^c^ |
| HDL-C (mmol/L), mean (median, IQR) | 1.33 (1.27, 1.14-1.42) | 1.42 (1.33, 1.15-1.62) | 0.292^c^ |
| LDL-C (mmol/L), mean (median, IQR) | 2.73 (2.76, 2.28-3.27) | 2.57 (2.53, 2.15-2.83) | 0.294^b^ |

Comparisons performed by Student's t-test or Chi-Square test or Mann-Whitney U test. **P* < 0.05. IQR, interquartile range; BMI, body mass index; VASI, vitiligo area scoring index; TC, total cholesterol; TG, triglyceride; HDL-C, high-density lipoprotein cholesterol; LDL-C, low-density lipoprotein cholesterol. ^a^Chi-Square test. ^b^Student *t*-test. ^c^Mann-Whitney U test. **Supplementary Table S3.** Comparisons of serum fatty acids concentration between vitiligo patients and healthy controls

| Fatty acid (umol/L) | Vitiligo (n = 48) | Control (n = 28) |
| --- | --- | --- |
| Caprylic acid | 1.97 (1.63-2.49) | 2.14 (1.46-3.06) |
| Capric acid | 0.56 (0.40-0.80) | 0.60 (0.31-1.15) |
| Dodecanoic acid | 1.02 (0.64-1.38) | 0.89 (0.57-1.22) |
| Myristic acid | 24.63 (17.67-29.98) | 26.74 (22.64-30.49) |
| Palmitic acid | 156.57 (121.15-192.72) | 163.58 (133.66-191.23) |
| Palmitoleic acid | 20.92 (12.54-30.99) | 19.75 (14.37-30.41) |
| Palmitelaidic acid | 0.92 (0.52-1.18) | 0.80 (0.55-1.09) |
| Stearic acid | 90.55 (77.32-108.07) | 90.89 (81.43-120.74) |
| Oleic acid | 286.35 (197.77-369.24) | 276.95 (234.83-358.89) |
| Linoleic acid | 212.92 (160.62-281.22) | 242.31 (175.51-282.52) |
| Gamma-Linolenic acid | 1.38 (0.97-2.10) | 1.78（1.16-2.09） |
| Alpha-Linolenic acid | 20.36 (10.60-26.45)* | 14.90 (12.09-18.44) |
| Arachidic acid | 1.85 (1.43-2.18)* | 2.17 (1.44-4.44) |
| Arachidonic acid | 8.12 (6.71-10.39)** | 10.58 (8.60-11.58) |
| Eicosapentaenoic acid | 0.52 (0.31-0.87) | 0.56 (0.36-0.75) |
| Behenic acid | 0.29 (0.23-0.42)* | 0.42 (0.27-0.80) |
| Docosahexaenoic acid | 3.02 (2.45-4.26) | 3.61 (2.78-5.38) |
| Tetracosanoic acid | 0.36 (0.19-0.64) | 0.32 (0.17-0.53) |
| Nervonic acid | 0.43 (0.32-0.56) | 0.41 (0.34-0.55) |
| Total FA | 822.57 (647.19-1035.80) | 858.07 (702.83-1012.53) |
| n-3PUFA | 23.78 (14.20-32.35) | 20.45 (15.29-23.32) |
| n-6PUFA | 221.04 (167.73-290.31) | 256.14 (184.05-295.14) |
| n-6/n-3 ratio | 11.61 (7.92-13.29) | 12.82 (10.32-14.30) |
| SFA | 271.93 (230.07-338.83) | 294.44 (254.29-342.75) |
| MUFA | 311.03 (214.13-399.77) | 301.45 (248.41-368.66) |
| PUFA | 244.73 (193.44-330.33) | 278.58 (201.61-314.22) |
| %SFA | 0.33 (0.31-0.36) | 0.34 (0.32-0.37) |
| %MUFA | 0.37 (0.33-0.39) | 0.35 (0.33-0.38) |
| %PUFA | 0.30 (0.28-0.33) | 0.30 (0.28-0.32) |
| UFA | 561.70 (411.67-712.90) | 569.36 (462.78-695.74) |
| SFA/UFA | 0.49 (0.46-0.55) | 0.52 (0.47-0.59) |

Comparisons performed by one-way ANOVA or Mann-Whitney *U* test. FA, fatty acids; SFA, saturated fatty acids; PUFA, polyunsaturated fatty acids; MUFA, monounsaturated fatty acids; UFA, unsaturated fatty acids; ANOVA, analysis of variance. Data are presented as median and interquartile range (IQR). * *P* < 0.05, ** *P* < 0.01.

**Supplementary Table S4.** Binary logistic regression of risk factor for vitiligo.

| Risk factor | Regression coefficient | Standard error | Wald χ^2^ value | *P* value | OR | 95% CI of OR | |
| --- | --- | --- | --- | --- | --- | --- | --- |
|  |  |  |  |  |  | Lower | Upper |
| ALA | 0.108 | 0.043 | 6.42 | 0.011 | 1.114 | 1.025 | 1.212 |
| AA | -0.782 | 0.371 | 4.431 | 0.035 | 0.458 | 0.221 | 0.948 |
| ARA | -0.391 | 0.134 | 8.509 | 0.004 | 0.677 | 0.52 | 0.88 |
| BA | 0.24 | 0.864 | 0.077 | 0.781 | 1.271 | 0.234 | 6.909 |

ALA, α-linolenic acid; AA, arachidic acid; ARA, arachidonic acid; BA, behenic acid.

**Supplementary Figures and Figure Legends**


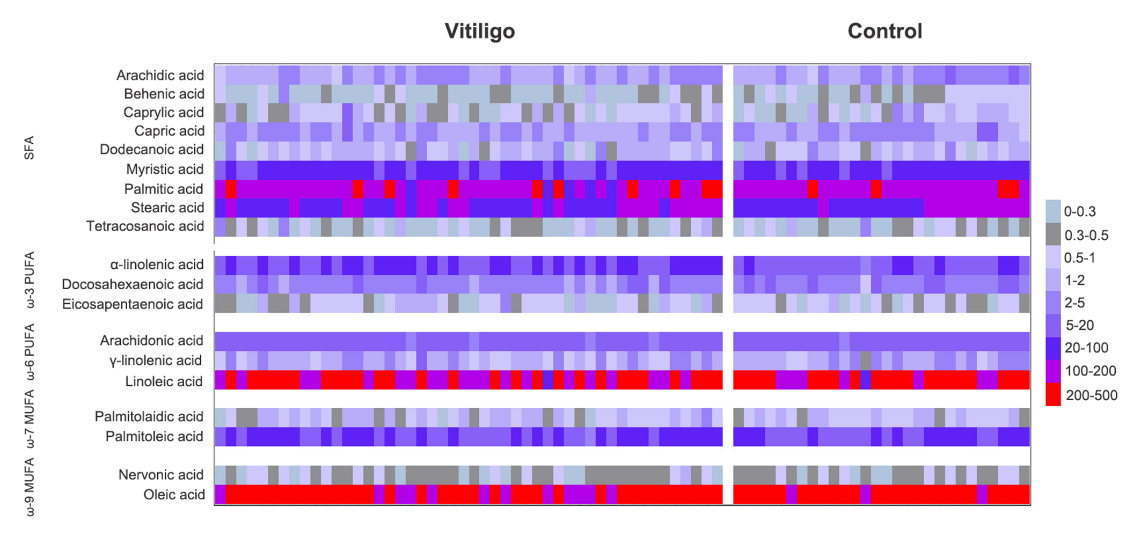


**Supplementary Figure S1** Heatmap of serum fatty acids identified in vitiligo patients and healthy controls. Heatmap represents serum concentrations of different fatty acids based on targeted metabolomics scan of 48 vitiligo patients and 28 healthy participants. Color plot is based on the different levels of the metabolites within cohort study and showed as concentrations. SFA, saturated fatty acid; PUFA, polyunsaturated fatty acid; MUFA, monounsaturated fatty acid.


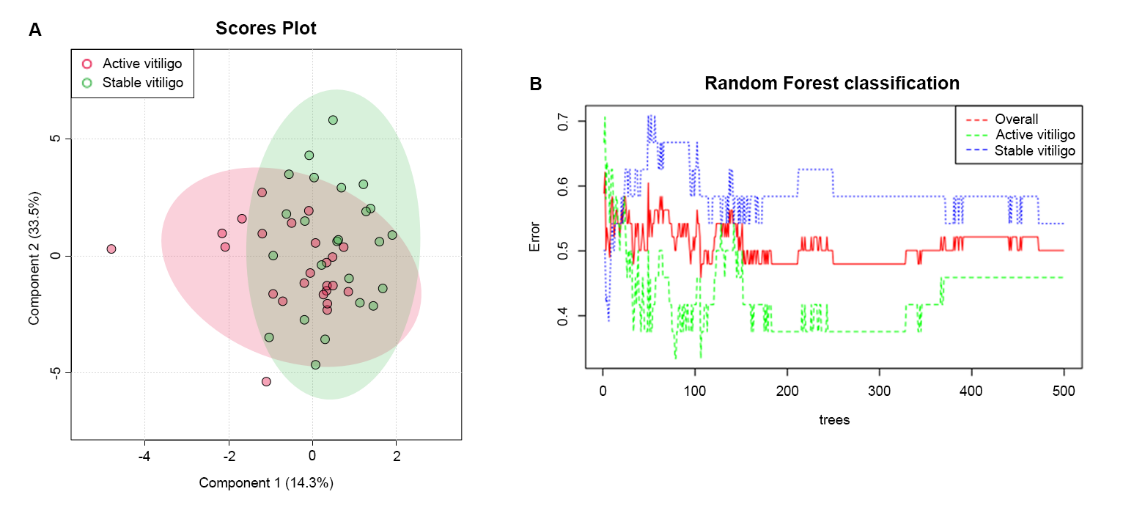


**Supplementary Figure S2** Utilization of fatty acid profiles for classification of active vitiligo and stable vitiligo by PLS-DA and RF analysis. (A) Diagram of PLS-DA model. (B) Cumulative error rate of RF classification. The overall error rate is shown as the red line, the green and blue lines represent the error rate for active vitiligo and stable vitiligo, respectively. PLS-DA, partial least squares discriminant analysis; RF, random forest.


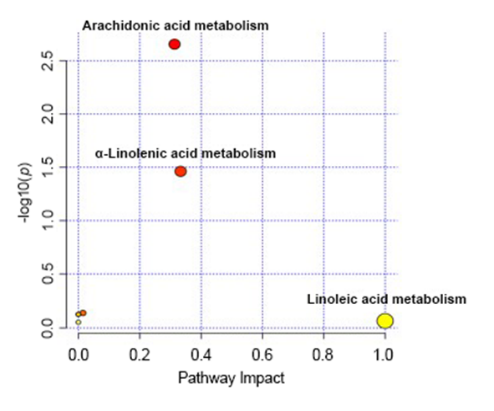


**Supplementary Figure S3** The pathway impact plot based on 19 fatty acids by pathway analysis using MetaboAnalyst 5.0. Redder colors describe lower *p*-values, and larger circles describe higher impact factors. The significantly changed pathway along with low *p*-values and large pathway impact factors. The pathways were prominently enriched in arachidonic acid metabolism, alpha-linolenic acid metabolism and linoleic acid metabolism.


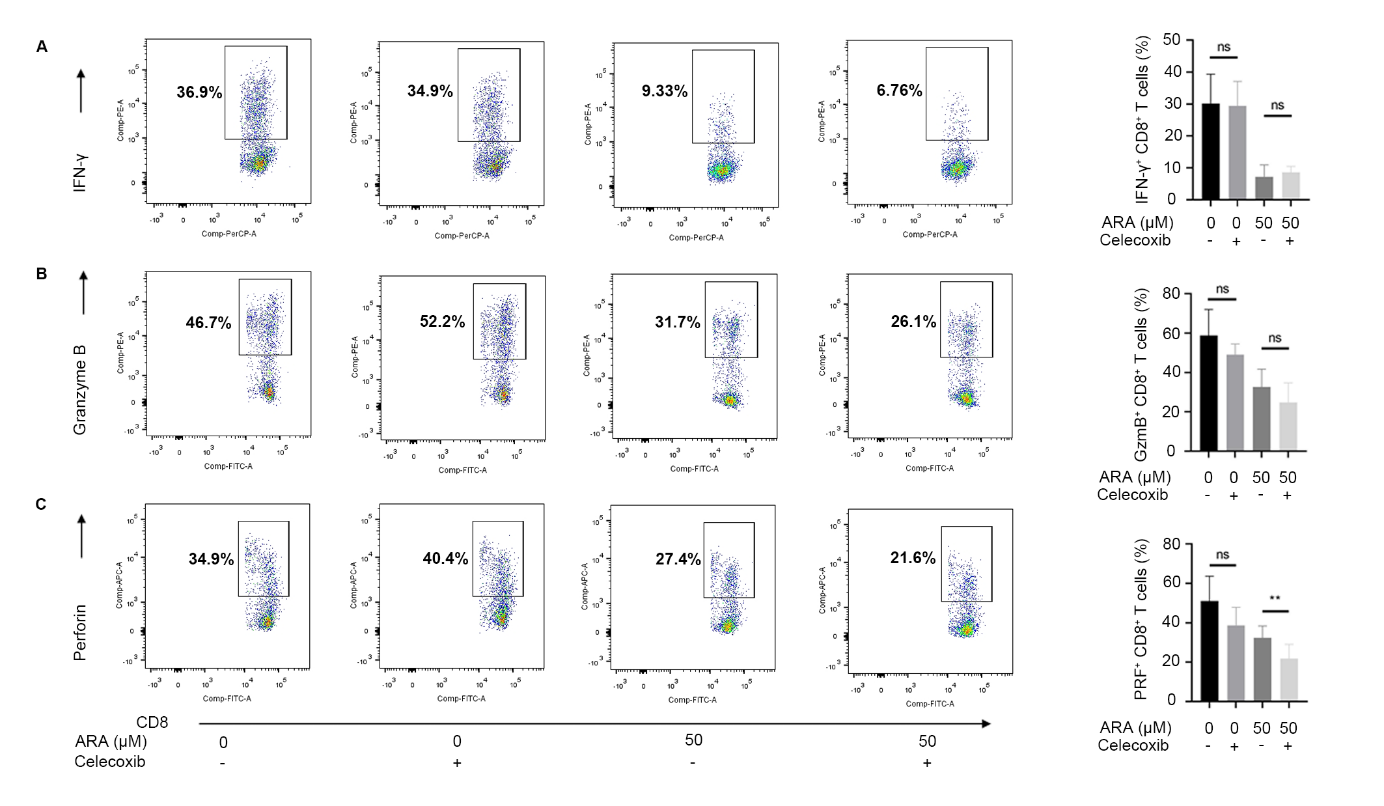


**Supplementary Figure S4** COX-2 inhibitors had no effect on CD8^+^ T cell activation and effector functions. Vitiligo PBMCs were stimulated with anti-CD3/CD28 for 48 hours in different conditions (with or without celecoxib (1μM) in the presence or absence of ARA (50μM)). FACS quantification for percentage of CD69^+^ CD8^+^ T cells intotal CD8^+^ T cells. Expression of IFN-γ (A), Granzyme B (B), and Perforin (C) in CD8^+^ T cells was determined by flow cytometry. μM, μmol/L, ns, no significance, * *P* < 0.05, ** *P* < 0.01.
